# Contraceptive Use and the Poor: A Matter of Choice?

**DOI:** 10.1371/journal.pmed.0040049

**Published:** 2007-02-06

**Authors:** Duff Gillespie

## Abstract

Gillespie discusses a new study showing that, despite the overall increase in the use of modern contraceptives, use among the poor remains quite low compared with wealthier segments of society.

From the “Health for All” declaration made at Alma Alta in 1978 to the recently created Commission on Social Determinants of Health, a recurrent theme is health equity, whereby all segments of society should have equal access to and reap the benefits of health-enhancing interventions [1,2]. Since 1978, a substantial literature has addressed health equity, much of it relating to the gap between the high rhetorical priority given health equity and its programmatic neglect [3]. Contrastingly, much less has been written about equity in family planning. The important article by Gakidou and Vayena in this issue of *PLoS Medicine* goes a long way toward filling this imbalance in attention [4].

Using Demographic and Health Surveys data from 55 developing countries, Gakidou and Vayena show that despite the overall increase in the use of modern contraceptives, the use among the poor remains quite low compared to the better-off segments of society. This difference in use rates among different groups is well known. While there is much to recommend this article, the authors' two main contributions is their documentation that the contraceptive use gap has persisted over time despite increases in gross domestic product (GDP) and showing the important role family planning services availability can play in closing this gap.

## “Socioeconomic Development is the Best Contraceptive”

It is not clear whether John D. Rockefeller III actually uttered the aphorism, “Socioeconomic development is the best contraceptive” at a 1974 UN population conference in Bucharest. However, he did deliver a speech whose message still resonates today and which has framed how contraceptive use among the poor is considered [5,6]. Rockefeller, the founder of the Population Council and, until this meeting, a strong advocate of “population control” through organized family planning programs, recanted such an approach and basically said people's socioeconomic status, especially women's, must improve before they will want to limit their families. Over the last three decades, much energy has been

The contraceptive use gap has persisted over time despite increases in GDP

expended over the relative importance of socioeconomic characteristics and service availability in predicting persons' reproductive behavior [6]. One of the hallmarks of equity is that something must be desired before its absence can be considered an inequity. When considering health, it is safe to assume that persons want to live and be healthy. If they are ill or dying at a greater rate than the better-off, one can presume they find their disadvantaged state undesirable and, therefore, it is an inequity [7]. Appling the equity concept to fertility behavior is much more difficult. While death and illness are always undesirable, pregnancy is sometimes desired, sometimes not.

Marmot has pointed out that attributing differences in health behavior and status to socioeconomic factors is risky business [8]. Indeed, his discussion highlights how little we know about differences in health found within and between countries. Gakidou and Vayena confirm Marmot's wisdom. If socioeconomic development were important for increasing the desire for smaller families and, hence, greater contraceptive use, one would like to see at least a modest correlation between increased GDP and a narrowing of the contraceptive use gap among the poor and the better off. Gakidou and Vayena did not find such a correlation and, if anything, found just the opposite, i.e., an increase in the contraceptive use gap was associated with an increase in GDP. Gakidou and Vayena's finding does not disprove Rockefeller's maxim since, by definition, the poor have not benefited as much as the nonpoor from increases in GDP and, therefore, cannot be expected to have as much a change in their fertility desires and behavior as their wealthier counterparts. The poor did increase their contraceptive use, but not as much as the rich. The authors also note that increases in GDP enable countries to improve the poor's access to family planning services, something they believe has not happened.

Another example of how Gakidou and Vayena's study highlights our limited understanding of the dynamics of inequity is their finding that, despite an increase of contraceptive use across all groups, the gap between the rich and the poor increased as GDP increased. This finding does not support Hart's classic hypothesis that the needs of the poor will not be met until those of the better-off segments are satiated [9]. If Hart were correct, one would expect a narrowing of the equity gap.

## Do the Poor Want Family Planning Services?

There is no doubt that socioeconomic status influences fertility desires and behavior. The real equity issue is whether the differences in contraceptive use among the poor and rich are explained by their different socioeconomic situations. Is the lower use of modern contraceptives among the poor due to a lack of desire for contraceptives? Or is it because they do not have access to family planning services? Although this study does not directly address these questions, the authors did find that access to family planning services (using the proxy of skilled birth attendance) was strongly associated with contraceptive use among the poorer segments of society. One could propose that the entire demand for family planning has been satisfied among the poor, something Gakidou and Vayena do not explore. However, a recent analysis of Demographic and Health Surveys data that did explicitly explore the relationship between demand for family planning and access to services found that in most countries family planning services were not meeting the demands of the poor [10], which supports Gakidou and Vayena's recommendation that making family planning services more available to the poor is critical for narrowing the poor–rich contraceptive use gap.

## Filling the Knowledge Gap

Calls to close the poor–rich health gap will not and cannot be effectively answered until our understanding of health inequities is greatly increased. There are examples of successful projects closing the inequity gap, but these have been of limited scope and short duration [3]. We really do not yet know what works. Even more sobering, we cannot adequately describe, much less explain, health inequities. Data availability frames how we describe and what we analyze. For the developing world, the boundaries of equity are largely defined by the Demographic and Health Surveys [11]. This lack of data has contributed to the lack of consensus on nomenclature and measurements [12]. A recent review of equity research by Oxman and colleagues found that most studies have been nested in larger studies or programs that are not primarily concerned with equity [13]. The clarion calls of governments and organizations to close the health equity gap will remain unanswered and unanswerable without investments in research to inform policy makers and to guide program administrators on what can and should be done to close the inequity gap.

## Abbreviations

GDP: gross domestic product
